# The association of tumor-expressed REG4, SPINK4 and alpha-1 antitrypsin with cancer-associated thrombosis in colorectal cancer

**DOI:** 10.1007/s11239-023-02907-6

**Published:** 2023-12-08

**Authors:** Jeroen T. Buijs, Robin van Beijnum, Rayna J. S. Anijs, El Houari Laghmani, Lily Sensuk, Cas Minderhoud, Betül Ünlü, Frederikus A. Klok, Peter J. K. Kuppen, Suzanne C. Cannegieter, Henri H. Versteeg

**Affiliations:** 1 Einthoven Laboratory for Vascular and Regenerative Medicine, Division of Thrombosis and Hemostasis, Department of Internal Medicine, Leiden University Medical Center, Leiden, The Netherlands; 2 Department of Surgery, LUMC, Leiden, The Netherlands; 3 Department of Clinical Epidemiology, LUMC, Leiden, The Netherlands; 4 Einthoven Laboratory for Vascular and Regenerative Medicine, Division of Thrombosis and Hemostasis, Department of Internal Medicine, Leiden University Medical Center, P.O. Box 9600, 2300 RC Leiden, The Netherlands

**Keywords:** Venous thrombosis, Thromboprophylaxis, Cancer-associated thrombosis, Alpha-1 antitrypsin

## Abstract

Novel biomarkers are needed to improve current imperfect risk prediction models for cancer-associated thrombosis (CAT). We recently identified an RNA-sequencing profile that associates with CAT in colorectal cancer (CRC) patients, with *REG4*, *SPINK4*, and *SERPINA1* as the top-3 upregulated genes at mRNA level. In the current study, we investigated whether protein expression of REG4, SPINK4 and alpha-1 antitrypsin (A1AT, encoded by *SERPINA1*) in the tumor associated with CAT in an independent cohort of CRC patients. From 418 patients with resected CRC, 18 patients who developed CAT were age, sex, and tumor stage-matched to 18 CRC patients without CAT. Protein expression was detected by immunohistochemical staining and scored blindly by assessing the H-score (percentage positive cells*scoring intensity). The association with CAT was assessed by means of logistic regression, using patients with an H-score below 33 as reference group. The odds ratios (ORs) for developing CAT for patients with A1AT^high^, REG4^high^, SPINK4^high^ tumors were 3.5 (95%CI 0.8–14.5), 2.0 (95%CI 0.5–7.6) and 2.0 (95%CI 0.5–7.4) when compared to A1AT^low^, REG4^low^, SPINK4^low^, respectively. The OR was increased to 24.0 (95%CI 1.1–505.1) when two proteins were combined (A1AT^high^/REG4^high^). This nested case–control study shows that combined protein expression of A1AT and REG4 associate with CAT in patients with colorectal cancer. Therefore, REG4/A1AT are potential biomarkers to improve the identification of patients with CRC who may benefit from thromboprophylaxis.

**Supplementary Information:**

The online version contains supplementary material available at 10.1007/s11239-023-02907-6.

## Highlights

- Cancer patients are at increased risk of developing venous thromboembolism (VTE).
- Better biomarkers are needed to predict VTE in cancer patients.
- At mRNA level, tumor-expressed *REG4*, *SPINK4* and *SERPINA1* associate with VTE.
- The gene *SERPINA1* encodes for Alpha-1 antitrypsin (A1AT).
- Combined REG4/A1AT protein expression associates with VTE in an independent cohort.

## Introduction

*Venous thromboembolism* (VTE) is a common cardiovascular disease worldwide, with an estimated incidence rate of 3 per 1000 person years [1]. VTE is linked to increased mortality and morbidity and drastically reduces the *quality of life* [1–3]. One of the major risk factors for VTE is cancer, with an average ninefold increased risk of developing VTE in the 1st year after cancer diagnosis [1].

Colorectal cancer (CRC) is the third most common cancer worldwide, with more than 1.9 million cases diagnosed yearly, and an ever rising incidence. As patients with colon and rectal cancer have a moderately high risk for VTE (incidence rates of 36 and 32.9, per 1000 person years [1]), CRC presents as a fast-rising *disease burden* globally.

Although thromboprophylaxis effectively reduces the incidence of thrombosis in cancer patients, its routine use in all cancer patients is not recommended, due to the high number to treat and increased risk of (fatal) bleeding in a population that is already at increased risk for bleedings [4]. The threshold to consider thromboprophylaxis in ambulatory cancer patients has lowered with the possibility to prescribe direct oral anticoagulants (DOACs) [4]. Even so, the optimal selection of patients who may benefit from thromboprophylaxis is still debated [5, 6].

Several risk prediction models have been developed to identify patients with cancer at high risk of developing VTE who benefit most from *thromboprophylaxis*. The Khorana score is the cancer–associated thrombosis (CAT) risk assessment model currently recommended in a clinical setting, and includes *cancer type*, *body mass index*, and blood parameters such as hemoglobin level, platelet, and leukocyte counts [4, 7]. Despite clinical recommendation, the Khorana score performed suboptimally in external validation studies [8]. One of the reasons for the suboptimal performance may be that the Khorana score was designed to select high-risk ambulatory patients undergoing chemotherapy, whereas many validation studies used other inclusion criteria, such as chemo-naïve patients, or included a different distribution of tumor types. A large meta-analysis comprising 54 studies showed that patients with a high Khorana score had only a 1.6-fold higher risk of developing VTE when compared to those with a low Khorana score [8].Therefore, there is an urgent need to identify novel biomarkers that alone or by incorporating into existing risk models, can better select patients that would benefit most from thromboprophylaxis.

To discover, in an unbiased approach, novel tumor-expressed genes that associate with CAT, we have previously performed next-generation RNA-sequencing (RNA-seq) of laser capture microdissected tumor cells from CRC patients [9]. By comparing tumors from patients that developed CAT before CRC diagnosis with patients that did not develop CAT, we found that the three most upregulated genes were *SERPINA1*, *REG4* and *SPINK4*.

*REG4* and *SPINK4* have been shown top regulated genes in inflammatory bowel disease (IBD), but with no clear role in development of VTE [11]. Nonetheless, *SERPINA1* encodes for the protein A1AT, which binds and neutralizes activated protein C (APC), a serine protease that proteolytically inactivates the activated coagulation co-factors Va and VIIIa [18].

In the current study, we determined whether the top-3 differentially regulated genes (*SERPINA1*, *REG4* and* SPINK4*), are increased at protein level in an independent and larger cohort of CRC patients.

## Methods

### Study design

In this study we retrospectively identified patients with CRC in the hospital’s administrative’s system, and assessed the primary event of the study, the development of CAT. Subsequently, we performed immunostainings to study the level of expression of three predefined proteins in the tumor in a nested case–control setting. Immunohistochemical stainings of tumor samples capture a wealth of information, demonstrating the intensity, cellular location and distribution of the protein of interest. On the other hand, immunohistochemical stainings are a relatively work-laborious process. For these reasons, we have chosen the nested case–control study design, which—compares to a cohort study—requires comparatively fewer subjects.

### Patients

**Fig. 1:**
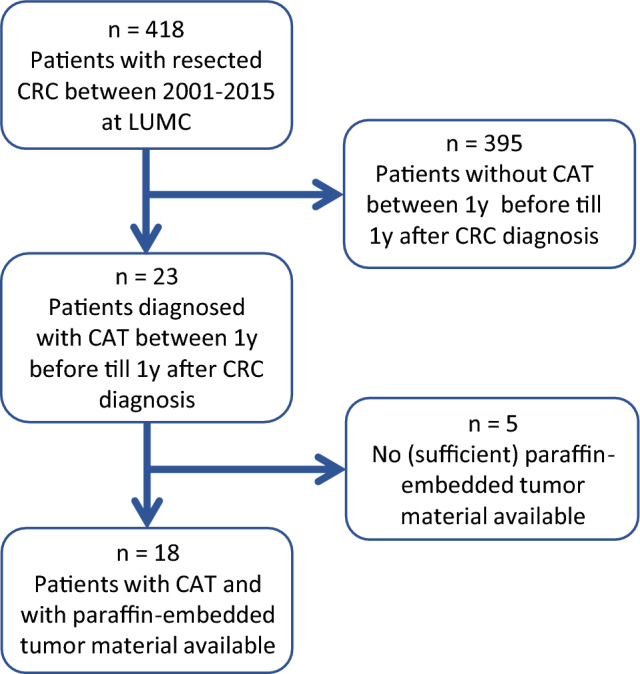
Flow diagram of colorectal cancer patients included in study. *LUMC* Leiden University Medical Center, *1y* 1 year, *CRC* colorectal cancer, *CAT* cancer-associated thrombosis, *CRC* colorectal cancer

In this study, 418 CRC patients were identified who underwent curative or palliative surgery between January 2001 and December 2015 at the Leiden University Medical Center (LUMC). From the hospital records, we assessed the variables age, gender and tumor stage, and the outcome variable CAT in the period 1 year before until 1 year after the date of CRC diagnosis. In this cohort 23 patients (5.5%) developed CAT. To ensure that there is enough material left for patient-related requests, paraffin blocks with potentially less than 200 µm of tumor tissue may not be used for research purposes at the department of Pathology, LUMC. Based on the unavailability of (sufficient) formalin fixed paraffin-embedded (FFPE) tumor tissue, 5 cases were excluded (Fig. 1). In a nested case–control setting, the 18 cases were individually matched based on sex, tumor stage and age, to 18 CRC control patients from the same study cohort that did not develop CAT. All data were manually collected from patients records by in-depth chart review by a medically trained data collector and pseudonymized. All thromboembolic events were adjudicated by an independent expert. This study was approved by the local institutional review board (the Medical Ethics Research Committee Leiden The Hague Delft) (#G20.062) and performed under guidelines of good clinical practice. The need for informed consent was waived by the institutional review board due to the retrospective study design and the fact that the majority of the patients were deceased at the start of the study. STROBE guidelines for reporting of observational studies were followed.

### Immunohistochemistry

Five µm sections were cut from FFPE tumors, mounted on silane-coated adhesive slides (Starfrost, Knittel, Germany), dried overnight at 37 °C, and stored at 4 °C. At the start of the immunohistochemical staining, slides were pre-warmed at 37 °C for 10 min before deparaffinizing. Deparaffinizing and rehydration was performed according to the following series of steps: 5 min Histoclear (NationalDiagnostics, UK) (3×), 2 min 100% ethanol (2×), 2 min 96% ethanol, 2 min 70%, dH_2_O. Endogenous peroxidases in the tissue were blocked by incubation in 0.3% H_2_O_2_ in PBS for 20 min at room temperature. Hereafter, antigen retrieval was performed by boiling the slides for 10 min in 1 M citrate buffer (pH 6.0) in a microwave oven at full power (900 W). Slides were cooled on ice in the citrate buffer and washed in 0.5% Tween-20/PBS. Sections were encircled using a PAP pen (DAKO Agilent, USA) and blocked for 30 min with 2.5% normal goat serum (VECTOR, USA) for A1AT staining, 2.5% normal horse serum (VECTOR, USA) for REG4 staining or 5% bovine serum albumin for SPINK4 staining. Blocking solution was tipped off and sections were incubated overnight at 4 °C with primary antibody. Primary antibodies used were: REG4 antibody (polyclonal Goat IgG, dilution 1:400, R&D systems, AF1379, AB_2178705), SPINK4 antibody (rabbit polyclonal, dilution 1:400; Sigma-Aldrich, HPA007286, AB_1080083) and a rabbit polyclonal A1AT antibody (dilution 1:3000). Primary antibodies against A1AT were raised by immunizing rabbits with several rounds of injecting human A1AT protein. Of the 15 rounds of plasma collection, we used in this project antibodies that were collected from the 12th round of plasma collection. IgG primary antibody isotype controls from the same species were used as negative controls. After washing with 0.5% Tween-20/PBS, the sections were incubated for 45 min with horseradish peroxide-labelled secondary antibodies recognizing rabbit antibodies (DAKO Agilent, USA) for the A1AT and SPINK4 staining, and recognizing goat antibodies (VECTOR, USA) for the REG4 staining. Immunecomplexes were visualized using NovaRed (VECTOR, USA) according to manufacturer’s protocol, and tissues were counterstained with 1:4 diluted Mayer’s hematoxylin and rinsed in slowly running tapwater for 10 min. After washing in demineralized water tissues were air-dried and mounted using 1:1 xylene histomount (NationalDiagnostics, UK).

Representative pictures (Figs. 3, 4, 5) were taken using an Olympus BX51 microscope mounted with an XC30 color camera with U-TV1X-2/UCAM3 camera adapter using the lenses UPlanFL N 4×/0.13, UPlanFL N 10×/0.30, UPlanSApo 20×/0.75 and UPlanSApo lens 40×/0.95, and CellSens software (all Olympus Life Science).

### Antibody validation

**Fig. 2:**
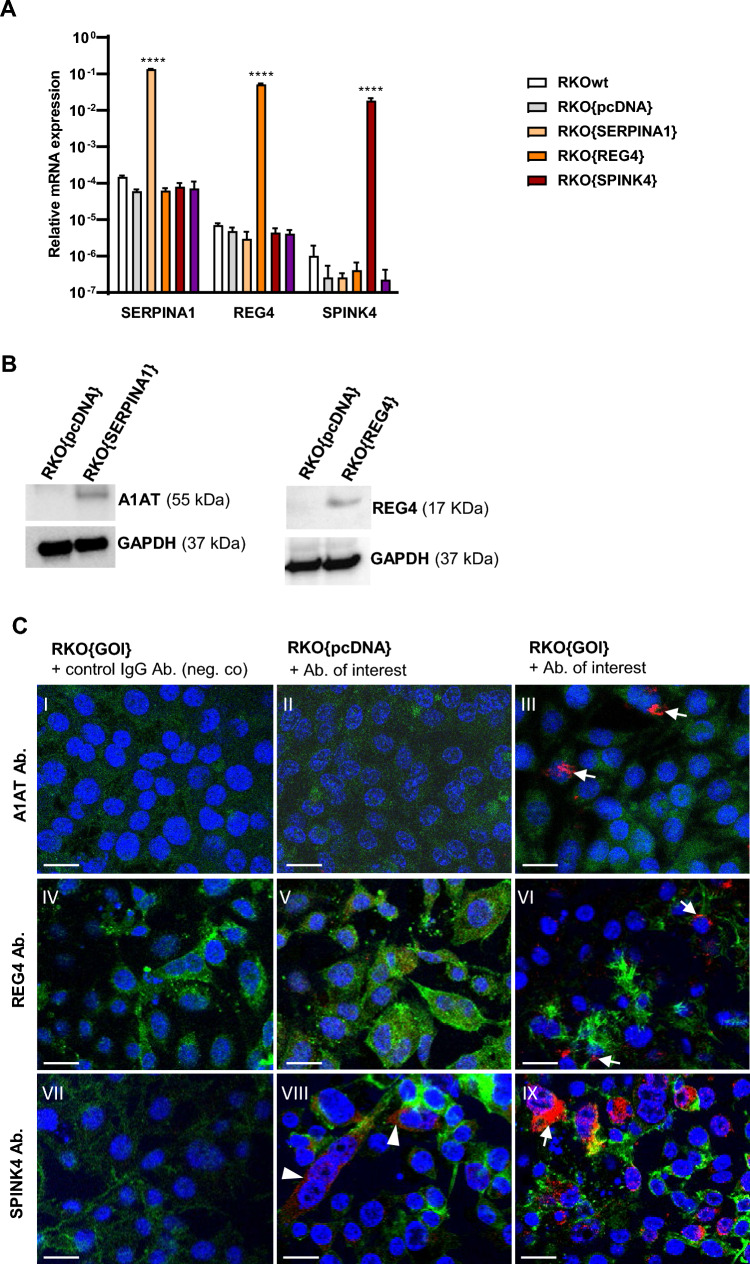
Immunofluorescence staining of RKO colorectal cancer cells overexpressing A1AT, REG4 or SPINK4 to validate antibodies used. **A** qPCR analysis showing mRNA expression levels in 2^-(((Ct GAPDH + Ct ACTB)/2) − Ct GOI). Per gene, the mean of each cell line was compared to all other cell lines using a one-way ANOVA with Tukey’s multiple comparisons test. ****p < 0.0001 vs. all other cell lines, **p < 0.01 vs. all other cell lines. **B** Western blot analysis showed bands at 55 kDa and 17 kDa for A1AT and REG4 respectively. **C** No A1AT staining is observed in RKO{pcDNA} cells (II), but A1AT protein expression is detected in RKO [24] cells using the rabbit polyclonal A1AT antibody (III, arrow). While RKO{pcDNA} cells hardly express REG4 (V), REG4 protein expression is detected in RKO{REG4} cells (VI, arrow). RKO{pcDNA} cells show low basal expression of SPINK4 (VIII, arrowhead), increased protein expression of SPINK4 was detected in RKO{SPINK4} cells using the rabbit polyclonal SPINK4 antibody (IX). Corresponding IgG control antibodies showed no staining (I, IV, VII). Sale bars: 18 µm

To test the specificity of the antibodies, RKO colorectal cells were stably transfected with SERPINA1, REG4 and SPINK4, which resulted in a 2225, 10,591, 71,368-fold upregulation compared to RKO{pcDNA} control cells, respectively (Fig. 2A). Western blot analysis showed bands at expected heights for A1AT and REG4 (Fig. 2B). NB. As specified on the commercial datasheet (Sigma-Aldrich, HPA007286), the SPINK4 antibody worked for immunohistochemistry (IHC) and immunofluorescence (IF) stainings, but not for WB (data not shown). All three antibodies (A1AT, REG4 and SPINK4, see above) showed strong immunofluorescence staining in the RKO cells that were stably transfected with the respective gene of interest, when compared RKO{pcDNA} control cells or the IgG control samples (Fig. 2C, I–XII). Experimental details on immunofluorescence staining, Western Blot and qPCR analysis are provided in Supplementary Methods.

### A1AT antibody optimization

**Fig. 3:**
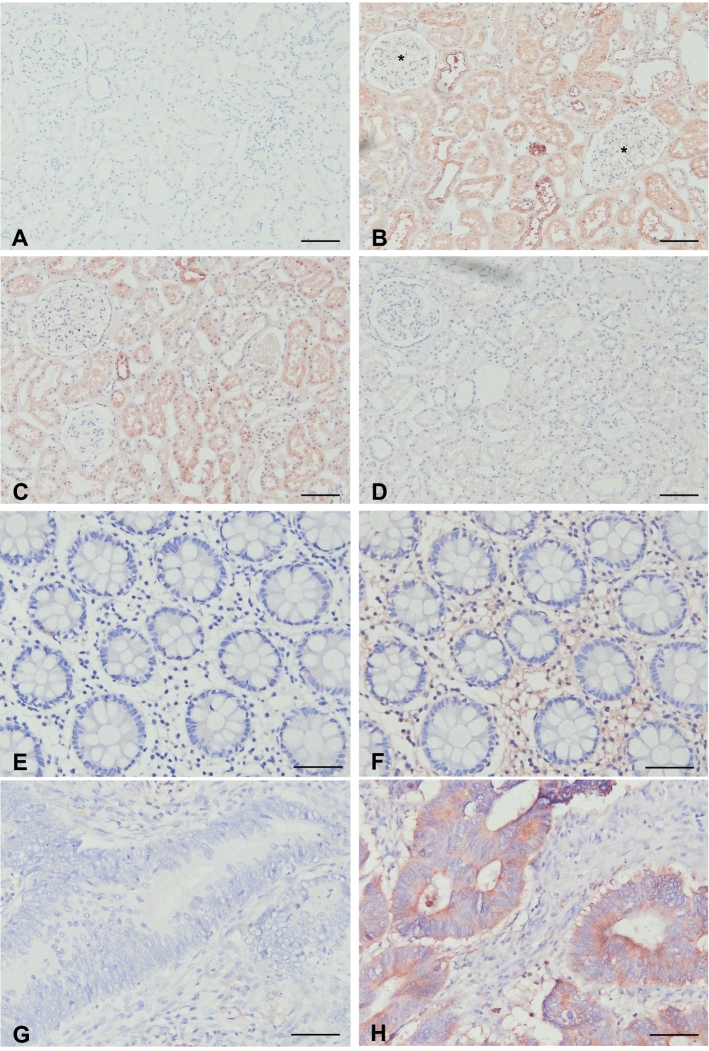
Immunohistochemical staining of alpha1-antitrypsin (A1AT). **A**–**D** Human kidney tissue was used to validate the custom A1AT rabbit polyclonal antibody. **A** Rabbit IgG isotype control antibodies were used as negative control, demonstrating a lack of aspecific binding in the protocol used. **B** A1AT (antibody diluted 1:3000) staining is observed in tubules, but not in glomeruli (*). **C**, **D** The A1AT antibody was 10 min pre-incubated with the A1AT protein, at an antibody:protein molar ratio of 1:1 (**C**) and 1:5 (**D**), demonstrating that A1AT staining could be completely blocked with the highest concentration A1AT protein added. **E**, **F** Adjacent non-cancerous colon tissue. **E** Rabbit IgG isotype control antibodies (negative control). **F** A1AT staining was not detected in epithelial cells of the crypts, while the stromal compartment was slightly positive. While some tumors had low or no detectable expression levels of A1AT (**G**), other tumors showed high expression of A1AT (**H**). Scale bars **A**–**D** 200 µm, **E**–**H** 50 µm

To optimize the A1AT immunostaining using the custom-made rabbit polyclonal antibody we used human kidney samples. Positive A1AT staining was observed in the tubules, but not in the glomeruli (Fig. 3A–D). This is in agreement with other A1AT immunostainings shown on ProteinAtlas using other A1AT antibodies (rabbit polyclonal antibodies HPA000927, HPA001292 and CAB013211, or the mouse monoclonal antibodies CAB016648 and CAB073396), demonstrating low or no expression of A1AT in glomeruli, and moderate A1AT staining of the tubules [10]. Furthermore, we found that pre-incubation of the A1AT antibody with human A1AT protein at a molar ratio 1:5 almost completely blocked the A1AT staining (Fig. 3D), underlining the specificity of the A1AT antibody for the human A1AT protein.

### Scoring

Scoring was based on staining intensity and number of positive tumor cells. Staining intensity was scored on a scale from 0 to 3, 0: no staining, 1: low, 2: medium, and 3: intense staining. The percentage of positive tumor cells was estimated from three representative fields of the tumor (×40 magnification). The H-score was calculated by multiplying the percentage of positive tumor cells with the staining intensity, totalling a score between 0 and 300. All samples were blinded prior to scoring. All stainings were scored by RvB. A random subset of slides were scored by JB to calculate interobserver variability (Suppl. Fig. 1).

### Statistical analyses

The Wilcoxon matched-pairs signed rank test was used to compare the means. Logistic regression analysis was performed to determine the odds ratio with corresponding 95% confidence intervals. In the primary logistic regression analysis, patients with an H-score below 33 were set as reference group. Patients with an H-score below 33 correspond to less than one third of the tumor cells that express low levels of protein. The significance level for all tests was set *P* < 0.05. The IBM SPSS Statistics 29 software was used to performed the statistical analyses.

## Results

### Study patients

In the study cohort of 418 CRC patients, 23 patients (5.5%) were diagnosed with CAT between 1 year before and 1 year after CRC diagnosis, of which 5 patients did not have (sufficient) paraffin-embedded tumor tissue. Of the remaining 18 patients, half (9/18) were diagnosed with acute pulmonary embolism (PE), 27.8% (5/18) developed a deep vein thrombosis (DVT), of which 1 was in the vena cava inferior and 4 in the legs, and 4 patients developed a CAT at other sites (vena porta, vena mesenterica, vena jugularis and vena ovarica). Fourteen patients (14/18, 77.8%) developed a CAT after CRC was diagnosed, and four patients before the CRC was diagnosed. Twelve patients were female, and the average age was 66.7 years.

### Study results

Strong staining of A1AT (H-score > 100) was detected in 52.8% of the tumors, while 36.1% of the tumors had low/no expression (H-score < 33) (Fig. 3E–H, Fig. 6A). The mean H-score of A1AT for patients with CAT was 142.3 compared with 61.2 for age, sex, and cancer stage-matched patients that did not develop CAT (*P* = 0.154, Table 1).

**Table 1:** Means of protein expression levels in relation to cancer-associated thrombosis

	H-score from pts with CAT Average (median)	H-score from pts without CAT Average (median)	*P* value^a^	Odds ratio (± 95% CI)
A1AT	142.3 (168.0)	84.6 (46.0)	0.154	3.5 (0.8–14.8)
REG4	71.7 (32.0)	61.2 (14.5)	0.712	2.0 (0.5–7.6)
SPINK4	58.0 (45.0)	56.6 (7.5)	0.744	2.0 (0.5–7.4)

*CAT* cancer-associated thrombosis, *pts* patients, *CI* confidence interval

^a^Wilcoxon matched-pairs signed rank test

^b^Patients with an H-score below 33 (H < 33)^b^ or below H33 served as reference group in the logistic regression analysis

**Fig. 4:**
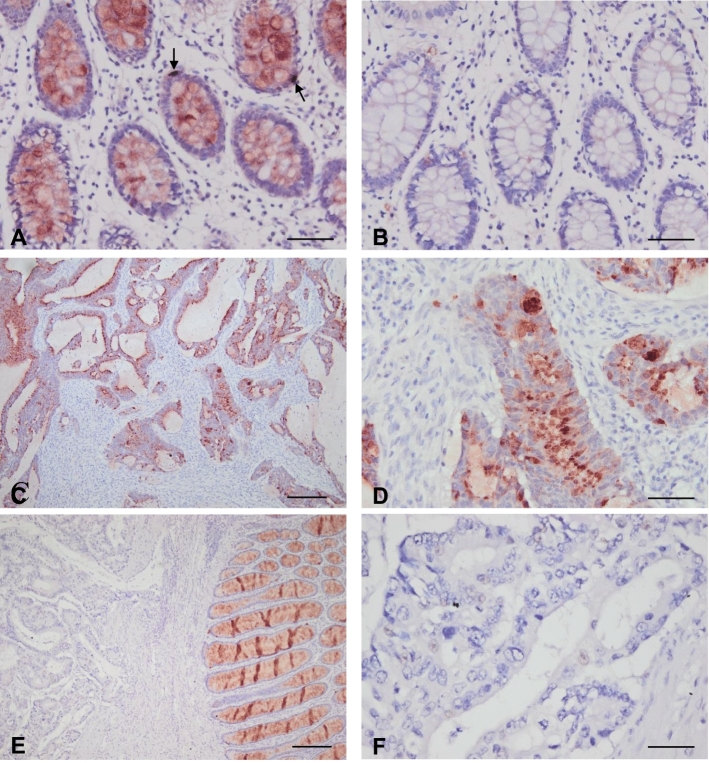
Immunohistochemical stainings of REG4. **A**, **B** Adjacent non-cancerous colon tissue. **A** REG4 immunostaining (antibody diluted 1:400) was observed in the colonic crypts. Intense immunostaining was observed in the secretory cells (arrows). **B** Goat IgG isotype controls were used as negative control, demonstrating a lack of aspecific binding of the protocol used. **C**, **D** Representative examples of a REG4-positive tumor with REG4-positive cancer cells, and REG4-negative stroma and stromal cells. **E**, **F** Representative examples of a REG4-negative tumor. **E** While the adjacent non-cancerous colonic crypts (right side of the image) are REG4-positive, the cancer cells (left side of the image) are REG4-negative. **F** A higher magnification of this tumor shows that none of the cancer or stromal cells are REG4-positive. Scale bars **A**, **B**, **D**, **F** 50 µm, **C**, **E** 200 µm

REG4 protein expression was detected in the normal crypts of adjacent non-cancerous colon tissue, with a very intense staining of the secretory cells and the stromal compartment completely negative (Fig. 4A–D). In the tumors, REG4 protein expression levels ranged from low/no expression (58.3% with an H-score < 33) to strong staining (25% with an H-score > 100) (Figs. 4E–H, 6B). The mean H-score of REG4 for patients with CAT was 71.7 compared with 61.2 for patients without CAT (*P* = 0.712, Table 1).

**Fig. 5:**
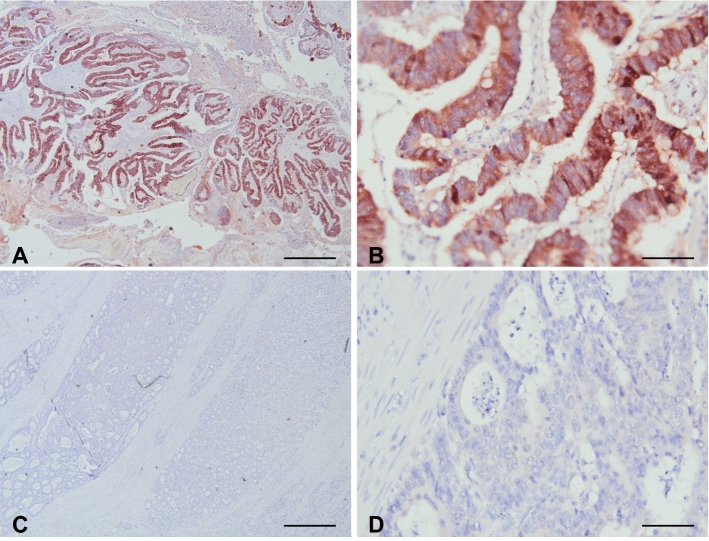
Immunohistochemical stainings of SPINK4. Representative examples of a SPINK4–positive tumor (**A**, **B**) and a SPINK4–negative tumor (**C**, **D**) (antibody dilution used 1:400). In the SPINK4–positive tumor (**A**, **B**), moderate SPINK4 staining was last detected in the stroma. Images on the right hand side (**B**, **D** scale bars: 50 µm) were taken with a 10× higher magnification than images on the left hand side (**A**, **C** scale bars: 500 µm)

**Fig. 6:**
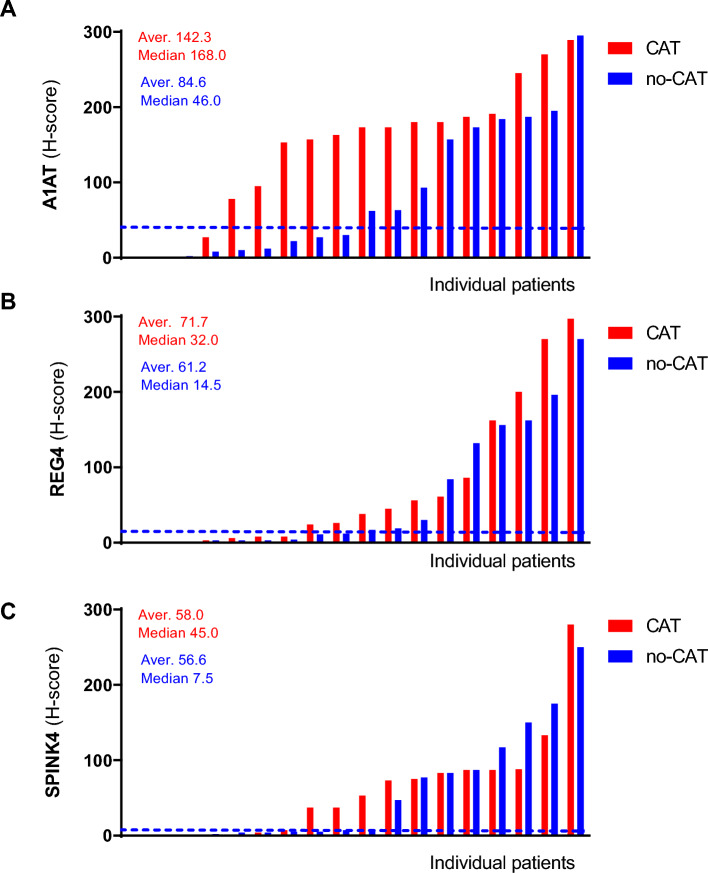
Waterfall plots immunohistochemical stainings. Waterfall plots of A1AT (**A**), REG4 (**B**) and SPINK4 (**C**) protein expression, in which each bar represents the H-score of the protein expression in the tumor of an individual patient. Blue dotted line, median value of control (‘no-CAT’)-group. *CAT* cancer-associated thrombosis

Low or no SPINK4 expression was observed in a relatively large proportion (47.2%) of the tumors (H-score < 33), and only 16.7% of the tumors demonstrated strong SPINK4 staining (H-score > 100) (Figs. 5A–D, 6C). No difference was observed in mean SPINK4 expression levels in patients with CAT (H-score: 58.0) compared with patients without CAT (H-score: 56.6) (*P* = 0.744, Table 1).

In the logistic regression analysis, patients with an H-score below 33 were set as reference group.

An H-score below 33 was used as threshold to define tumors that express low (or negative) levels of the protein of interest, as an H-score below 33 corresponds to less than one third of the tumor cells expressing low levels of the protein of interest. For logistic regression analysis we used the patients with an H-score below 33 as reference group for each protein. The OR for CAT for patients with tumors with strong staining of A1AT (A1AT^high^) was 3.5 (95% CI 0.8–14.8) compared with A1AT^low^ (Table 1). Patients with REG4^high^ and SPINK4^high^ tumors had ORs of 2.0 (95% CI 0.5–7.6) and 2.0 (95% CI 0.5–7.4), when compared with REG4^low^ and SPINK4^low^, respectively. The combination of A1AT with SPINK4 (A1AT^high^/SPINK4^high^), and particularly with REG4 (A1AT^high^/REG4^high^) resulted in increased ORs for CAT: 10.0 (95% CI 0.9–117.0) and 24.0 (95% CI 1.1–505.1) compared with patients with A1AT^low^/SPINK4^low^ and A1AT^low^/REG4^low^ tumors, respectively. Combining all three proteins (A1AT^high^/REG4^high^/SPINK4^high^) did not further increase the OR (OR 20.0, 95% CI 0.9–429.9) when compared with A1AT^low^/REG4^low^/SPINK4^low^ tumors (Table 2).

**Table 2:** Logistics regression of protein expression levels in relation to cancer-associated thrombosis

	Odds ratio (± 95% CI)^a^
A1AT^high^/REG4^high^	24.0 (1.1–505.1)
A1AT^high^/SPINK4^high^	10.0 (0.9–117.0)
REG4^high^/SPINK4^high^	2.4 (0.5–11.0)
A1AT^high^/REG4^high^/SPINK4^high^	20.0 (0.9–429.9)

Patients with an H-score below 33 (H < 33)

^a^Served as reference group in the logistic regression analysis

## Discussion

Our main findings are that the combination of REG4, SPINK4 and A1AT protein expression associates with CAT in an independent cohort of patients with CRC. The data that REG4, SPINK4 and A1AT protein expression associate with CAT are in line with our earlier findings that *REG4*, *SPINK4* and *SERPINA1* were the top-3 upregulated genes at mRNA level that associate with CAT [9]. In the current study, the combined protein expression of REG4 and A1AT demonstrated the strongest association with CAT.

Expression of REG4 and SPINK4 in the tumor may indicate a proinflammatory status of the tumor. In addition to being among the 21 most upregulated genes in inflammatory bowel disease (IBD), *SPINK4* was the gene that most strongly co-expressed with *REG4* in IBD [11]. In our CRC cohort, protein expression levels of SPINK4 also significantly correlated with REG4 (P = 0.001, Pearson r: 0.5995, data not shown). As a result, it is not surprising that combining these co-expressed genes (REG4^high^/SPINK4^high^) in a logistic regression analysis hardly resulted in an increase in the OR (OR 1.8) for developing CAT when compared with REG4^high^ (OR 1.6) or SPINK4^high^ (OR 2.0) alone.

Blood coagulation and the immune system of higher organisms are closely intertwined. Virtually all solid tumors induce a local or systemic inflammatory state, which may contribute to development of CAT. Indeed, inflammation was one of the pathways associated with CAT in the RNA-seq pathway analysis in CRC [9]. These findings were recently extended to lung cancer in a gene set enrichment analysis of RNA-seq data, demonstrating upregulation of genes in the inflammation and complement pathway, besides upregulation of genes associated with the KRAS signaling pathway [12].

REG4 has been shown to associate with CRC progression [13, 14]. Zhu et al. demonstrated that *REG4* mRNA was increased in 40 CRC samples compared with paired adjacent normal mucosa and REG4 proteins levels assessed by immunohistochemistry associated with distant metastasis and disease-free and overall survival [14]. Furthermore, Oue et al. showed that REG4 immunostaining associated with tumor grade, liver metastasis and poor survival, and that serum REG4 levels were increased in stage IV, but not stage I–III, CRC patients, when compared with 151 healthy controls [13]. In the colon, REG4-positive deep crypt secretory cells serve as an epithelial niche for LGR5-positive stem cells [15]. REG4 upregulation in the tumor may create additional cancer stem cell (CSC) niches, facilitating the growth of the aggressive subpopulation of LGR5-positive CSCs [15, 16]. CSCs are associated with enhanced invasion and metastasis. Rather than inflammation, an alternative explanation for the potential link of REG4 with CAT, is that REG4 increases intravasation of thrombogenic CRC cells into the blood circulation.

Of the three genes identified by RNA-seq, a trend for an association with CAT on protein level was observed for A1AT. A1AT is a protease inhibitor that keeps the activity of a variety of enzymes—particularly trypsin and neutrophil elastase (NE)—under control [17]. Deficiency in A1AT leads to extensive and prolonged NE-induced degradation of elastin resulting in reduced lung elasticity and respiratory complications [17]. There are three potential pro-thrombotic effects described through which A1AT may contribute to CAT. Firstly, A1AT binds and neutralizes activated protein C (APC), a serine protease that proteolytically inactivates the activated coagulation co-factors Va and VIIIa [18]. Individuals with low levels of APC, or with some resistance to the effects of APC, are at increased risk for VTE [19, 20]. Secondly, A1AT inhibits the enzymatic function of NE, a protein secreted by neutrophils during inflammation. NE degrades cross-linked fibrin, and reduction of NE activity may therefore be suggested to impair clearance of blood clots. However, by degradation of the α-chain of fibrin, NE also reduces the stimulating effect of fibrin on plasminogen, and the net effect of NE does not appear to be fibrinolytic [21]. Thirdly, A1AT regulates fibronectin, which is covalently linked to fibrin during clot formation mediating platelet adhesion to collagen [22]. In a variety of cancers, the transcription factor Zinc finger protein SNAI1 induces an epithelial-to-mesenchymal transition (EMT) and a CSC-like phenotype. Immunohistochemical analysis of 528 CRC tumors demonstrated that not only SNAI1, but also A1AT protein expression levels were associated with tumor stage, lymph node metastasis and poor clinical outcome. Moreover, SNAI1 directly upregulated *SERPINA1* (encoding A1AT protein) expression by binding its promoter region. Remarkably, the pro-metastatic effects of SNAI1 and A1AT on invasion and migration were mediated by upregulation of fibronectin [23]. In addition, Chang et al. showed that A1AT facilitates assembly of pericellular levels of fibronectin, facilitating lung metastasis [24].

The transmembrane glycoprotein TF is under physiological conditions expressed by most non-endothelial cells [25, 26]. In cancer, TF expression is regulated by both specific oncogenes and environmental factors [27, 28] and shown to regulate primary growth and metastasis formation in a variety of cancer models [29]. Yu and coworkers reported that driver mutations in colorectal cancer (*KRAS* and *TP53*) resulted in overexpression of TF via MEK/mitogen-activated protein (MAPK) and phosphatidylinositol 3’kinase (PI3K) [30]. In line, Ades et al. showed that *KRAS* mutational status associated with VTE in patients with colorectal cancer [31]. Interestingly, REG4 expression was recently shown to be induced by *KRAS* mutation in colorectal cancer cells, and act as a driver of K-RAS-induced tumorigenic effects [32, 33]. Therefore, *KRAS* mutational status may be the underlying genetic cause of the observed association of REG4 with VTE. Whether REG4 is also a driver—or a bystander—of thrombogenic effects remains to be investigated.

In all CAT risk prediction models, including the Khorana score [7], the PROTECHT score [34], the CONKO score [35], the ONKOTEV score [36] and the Vienna CATS score [37], the site of primary tumor is an important determinant. Consequently the discriminatory power is decreased in studies focusing on a single tumor type, e.g., in stage II–III CRC patients [38]. If tailored risk prediction modelling becomes common practice in a group of patients with single tumor type, tumor type specific biomarkers are needed to restore the predictive power.

The main limitation of the current study is the relatively small sample size, which is particularly relevant when analyzing the combined expression of proteins in the logistic regression analysis. Patients with A1AT^high^/REG4^high^/SPINK4^high^ tumors demonstrated an OR of 20.0 for CAT when compared with patients with A1AT^low^/REG4^low^/SPINK4^low^ tumors, but also demonstrated a relatively wide 95% CI (0.9–429.9). Nonetheless the high OR for REG4^high^/SPINK4^high^/A1AT^high^ tumors confirms the previously established association of A1AT, SPINK4 and REG4 mRNA expression with CAT. In particular, the validation cohort used demonstrated that combined expression REG4 and A1AT is associated with increased risk for CAT.

A limitation of using immunohistochemical staining as a detection method is the poor clinical translation. In general, immunohistochemical stainings are work-laborious and the results may vary largely between laboratories. Of interest, REG4 and A1AT are secreted proteins, and it would be of great interest to assess REG4 and A1AT plasma levels, as an ELISA-based assay would have far better clinical applicability as a biomarker when compared to an immunostaining. Performing ELISA-based assays in a larger cohorts would not only show whether REG4 and A1AT are good CAT biomarkers in CRC, including other cancer types in the cohort would also show whether REG4 and A1AT would be applicable as CAT biomarkers in other cancer types as well.

Besides the use of CAT biomarkers, it would be of utmost interest from a scientific point of view to perform mechanistic studies in in vitro, in vivo and thrombosis-on-a-chip models to elucidate the underlying biological mechanisms how REG4, SPINK4, and A1AT link with CAT.

In conclusion, we have shown that particularly the combination of REG4 with A1AT in tumors associate with CAT in patients with colorectal cancer. Upon validation in a large cohort, these candidate biomarkers could risk stratify colorectal cancer patients for thromboprophylaxis.

### Supplementary Information

Below is the link to the electronic supplementary material.

Supplementary file1 (PDF 129 kb)

Supplementary file2 (PDF 185 kb)

## Data Availability

Not applicable.

## Abbreviations

A1AT: Alpha-1 antitrypsin
APC: Activated protein C
CAT: Cancer-associated thrombosis
CI: Confidence interval
CRC: Colorectal cancer
CSC: Cancer stem cell
DOAC: Direct oral anticoagulant
DVT: Deep vein thrombosis
FFPE: Formalin fixed paraffin-embedded
LUMC: Leiden University Medical Center
MAPK: MEK/mitogen-activated protein
NE: Neutrophil elastase
OR: Odds ratio
PE: Pulmonary embolism
PI3K: Phosphatidylinositol 3’kinase
RNA-seq: RNA-sequencing
VTE: Venous thromboembolism
