# Supplementary material for: The association of tumor-expressed REG4, SPINK4 and alpha-1 antitrypsin with cancer-associated thrombosis in colorectal cancer
Source: J Thromb Thrombolysis. 2023 Dec 8;57(3):370–80. doi: 10.1007/s11239-023-02907-6 (PMC10961291; doi:10.1007/s11239-023-02907-6)

Supplementary Figure S1 Interobserver variability. A second observer (JB) blindly assessed the A1AT (A), REG4 (B), SPINK4 (C) staining intensity by taken a random subset of 10, 36, 14 slides respectively.

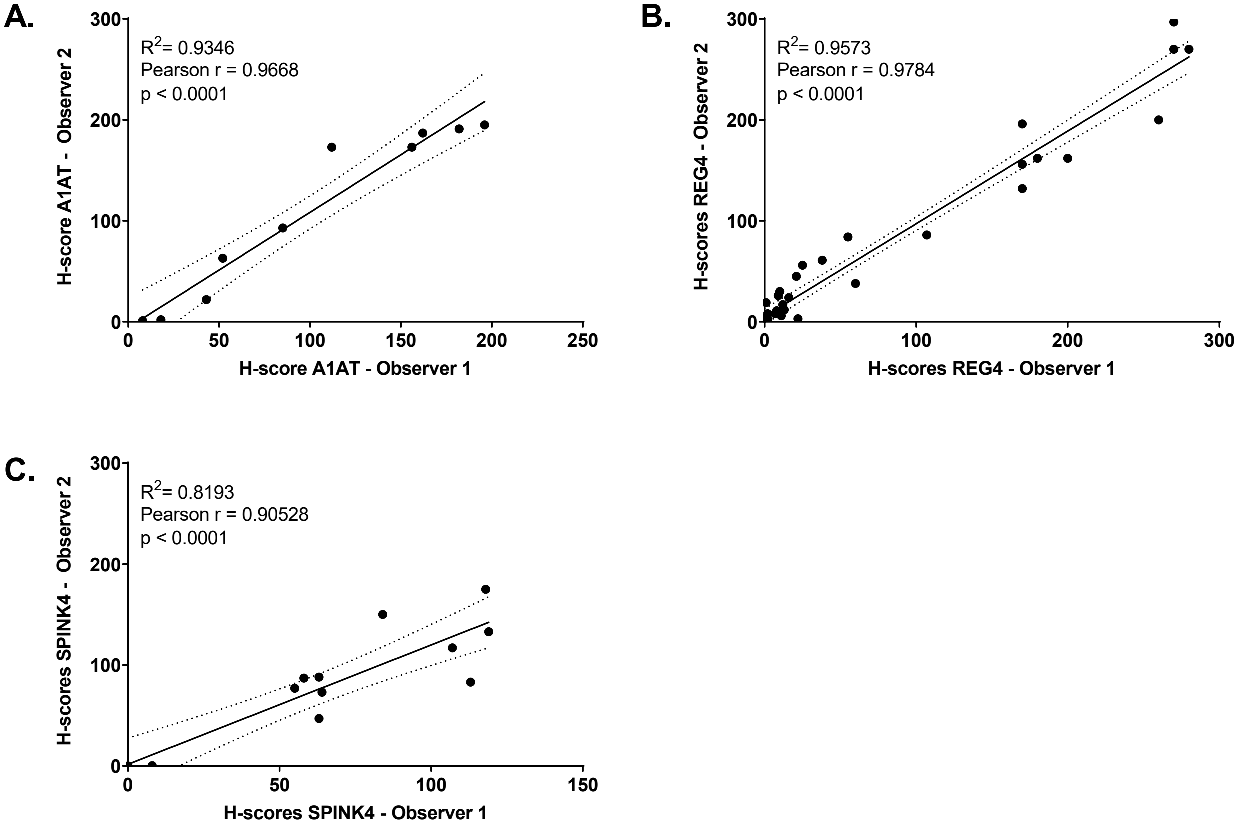

Supplement: Supplementary file 1 — Supplementary file1 (PDF 129 kb) [file 11239_2023_2907_MOESM1_ESM.pdf]
