# Supplementary material for: The association of tumor-expressed REG4, SPINK4 and alpha-1 antitrypsin with cancer-associated thrombosis in colorectal cancer
Source: J Thromb Thrombolysis. 2023 Dec 8;57(3):370–80. doi: 10.1007/s11239-023-02907-6 (PMC10961291; doi:10.1007/s11239-023-02907-6)
Supplement: Supplementary file 2 — Supplementary file2 (PDF 185 kb) [file 11239_2023_2907_MOESM2_ESM.pdf]

Supplementary Material & Methods

Confocal immunofluorescence microscopy

Glass coverslips were put in a 24-well and 250.000 RKO cells were seeded. After 3 days, cells were fixated for 15 minutes in 4% paraformaldehyde (PFA) (Alfa Aesar, Karlsruhe, Germany) (REG4, SPINK4) or 10 minutes in cold (-20ºC) methanol. Cells were washed in PBST (PBS/0.05% Tween-20), and PFA fixated cells were permeabilized with PBS/0.2% Triton X100 for 5 minutes. After washing in PBST, cells were blocked for 20 minutes with blocking buffer (1% BSA (Sigma-Aldrich), 5% FCS (Gibco) in PBS). Blocking buffer was removed and cells were incubate with first antibody diluted in blocking buffer for 45 minutes at room temperature. Primary antibodies used for the immunofluorescence staining of cells were the same as used in immunohistochemical staining of clinical material: A1AT rabbit polyclonal antibody (see main text), REG4 antibody (polyclonal Goat IgG, R&D systems, AF1379, AB_2178705), SPINK4 antibody (rabbit polyclonal; Sigma-Aldrich, HPA007286, AB_1080083) with respective dilutions of 1:100, 1:200, 1:100.

After washing in PBST, cells were incubated with the secondary antibodies - alexafluor-647 labelled Donkey anti-Rabbit (Invitrogen Molecular Probes) for the SPINK4 and A1AT antibodies or alexafluor-647 labelled Donkey anti-Goat (Invitrogen Molecular Probes) for REG4 antibody - diluted 1 to 750 in blocking buffer for 45 minutes in the dark. Subsequently, cells were incubated with HOECHST (Thermo Fisher Scientific) (diluted 1:10.000 in PBS) for 5 minutes at room temperature and fluorescein Isothiocynate-labelled phalloidin (Sigma-Aldrich) (diluted 1:500 in PBS) for 45 minutes at room temperature in the dark. Cover slips were mounted with ProLong® Diamond Antifade Mountant (Thermo Fisher Scientific) and put on an object glass, and dried overnight. Cells were visualized using the Leica TCS SP8 X WLL converted confocal microscope.

Cell culture

RKO cells were cultured in Dulbecco's Modified Eagle Medium (DMEM) (Gibco, United Kingdom) containing 10% Fetal Bovine Serum (FBS) (PAN Biotech, United Kingdom) and 1% Penicillin/Streptomycin (P/S) (Gibco, United Kingdom).

Stable transfection

The FuGENE® HD Transfection Reagent (Promega, United States of America) was used according manufacturer's recommended protocol with an optimized ratio of FuGENE® HD to DNA construct of 2.5:1. The genes REG4, SERPINA1, SPINK4 were cloned into a pcDNA3.1 vector under zeocin resistance. One day after seeding 500,000 RKO cells per 6-well, cells were transfected by adding a mixture of 5ul Fugene HD, 2ug DNA (pcDNA with gene of interest) in 100 µl medium. Three days later, cells were put on 125 µg/ml Zeocine (Invitrogen, United States of America).

RNA isolation

Cells in 6-well plate were washed with PBS and 1 ml of TRIsure (Bioline, Germany) was added. Subsequently, cells were scraped and transferred to Eppendorf tube. 220 µL chloroform (Merck KGaA, Germany) was added to the sample, followed by shaking the sample for 30 seconds. Samples were cooled on ice for 5 minutes and centrifuged at 4˚C and 14000 rpm for 15 minutes. Next, the transparent layer of the sample was transferred into a new 2 ml Eppendorf tube and 450 µL isopropanol (Merck KGaA, Germany) was added to the sample. This was followed by shaking the Eppendorf tube for 30 seconds, cooling the sample on ice for 15 minutes, and centrifuging the sample at 4˚C and 14000 rpm for 15 minutes. Supernatant was discarded, and the Eppendorf tube was dried upside-down, and the Eppendorf tube was carefully dried with tissue. This was

immediately followed by adding 750 µL of 75% ethanol to the samples and centrifugation at 4˚C for 14000 rpm for 15 minutes. Supernatant was discarded, the Eppendorf tube was dried upside-down, and the Eppendorf was carefully dried with tissue. Next, the sample was air-dried and incubated shortly at 55˚C until the pellet became transparent. For each 10 samples, 10 µL Recombinant RNasin Ribonuclease (RNase) Inhibitor (Promega Benelux B.V, The Netherlands) dissolved in 180uL RNAase free water (Fresenius Kabi, Netherlands) was made. From this mix, 15 µL (or 20 µL when the RNA concentration exceeds 2000 ng/µL) was added. Lastly, the sample was centrifuged shortly and the RNA concentration and nucleic acid purity (the 260/230 and 260/280 ratios) were determined by NanoDrop™ 1000 Spectrophotometer (Thermo Fisher Scientific). Sample were stored at -20ºC.

cDNA synthesis

1 µg RNA was dissolved in 10 µL RNAase free water. 2 µL oligo deoxythymine (dT) primer was added to the sample. The sample was shortly vortexed and centrifuged, and incubated at 70˚C for 10 minutes, followed by centrifuging the sample shortly. In between, a buffer mix was made that contained per sample: 4 µL 5x first strand buffer (Invitrogen, United States of America), 2 µL Dithiothreitol (DTT) (Invitrogen, United States of America), 1 µL deoxynucleotide triphosphates (dNTP) mix (Thermo Fisher Scientific, United States of America) and 0.5 µL Recombinant RNAse Inhibitor. After cooling on ice, 7.5 µL was added to the buffer mix and incubated at 42˚C for 2 minutes. In between all steps, samples were vortexed and centrifuged shortly. 1 µL SuperScript II Reverse Transcriptase (Invitrogen, United States of America) was added, and incubated at 42˚C for 60 minutes, and stored at -20ºC.

Real time quantitative PCR (qPCR)

Total volumes of 10 µL per well were used in a 384-well plate, containing 5 µL of SYBR Select Master Mix (Thermo Fisher Scientific, United States of America), 4.2 µL of RNase free water, 0.2 µL of the forward primer and reverse primer (10 µM)(see below, Suppl. Table 3), and 0.4 µL cDNA. Next, the plate was sealed with a MicroSeal 'B' seal Seals (Bio-Rad Laboratories, United Kingdom). After centrifuging of the plate at 1800 rpm for 1 minute, the qPCR was run on the Bio-rad CFX (Bio-Rad Laboratories, United Kingdom) as follows: 2 minutes at 50˚C and 95˚C, and 40 cycles of 10 seconds at 95˚C and 1 minute at 60˚C, followed by performing a melt curve analysis at 60˚C to 95°C, with increment of 0.5°C for 15 seconds. Ct values were assessed using Bio-Rad CFX Manager Software, version 3.1 (Bio-Rad Laboratories).

Gene	Accession number	Exon(s)	Product size		Primer sequence
SERPINA1	NM_000925.5	2	91	*Forward*	ATGCTGCCCAGAAGACAGATA
				Reverse	CTGAAGGCGAACTCAGCCA
REG4	NM_032044.4	2-3	96	*Forward*	CTGCTCCTATTGCTGAGCTG
				Reverse	GGACTTGTGGTAAAACCATCCAG
SPINK4	NM_014471.3	1-3	100	*Forward*	CAGTGGGTAATCGCCCTGG
				Reverse	CACAGATGGGCATTCTTGAGAAA
GAPDH	NM_002046	4-6	175	*Forward*	TTCCAGGAGCGAGATCCCT
				Reverse	CACCCATGACGAACATGGG
ACTB	NM_001101	4-5	275	*Forward*	CAAGAGATGGCCACGGCTGCT
				Reverse	TCCTTCTGCATCCTGTCGGCA

Suppl. Table 3. Primer sequences

Western blot

Three days after seeding 250,000 cells per 6-well, 200 µL Sample Buffer Tris-Glycine SDS (Novex, United States of America) was added, cells were scraped and collected. To reduce viscosity, the sample was sonicated for 10 seconds by Soniprep 150 (Beun De Ronde, The Netherlands). Next, the samples were heated at 95°C for 5 minutes, followed by cooling on ice and measuring protein concentration with the NanoDrop.

Gel electrophoresis was performed using 12 or 15-well Bolt™ 4-12% Bis-Tris Plus Gels (Invitrogen, United States of America) and placed in the Blot Mini Gel Tank (Novex, United States of America) , which was filled with MES buffer. Gel was loaded with either 5 µL of marker (SeeBlue Plus2 Pre-stained Protein Standard; Thermo Fisher Scientific, United States of America) or 20 µL sample, followed by running the gel at 165V for 35 minutes. Subsequently the gel was transferred on a PVDF membrane, stored in Trans-Blot Turbo™ Mini PVDF Transfer Packs (Bio-rad Laboratories, United States of America). To blot the membrane, the gel on the PVDF membrane was brought into the cassette of the Trans-Blot Turbo system (Bio-Rad Laboratories, United States of America) and the Turbo program was run for 7 minutes. Next, the membrane was cut if necessary and blocked with 5 ml blocking buffer in a 50 ml tube, containing 2.5 g milk powder (Nutricia Protifar) dissolved in 50 ml MilliQ, for 60 minutes. All incubation, and washing steps, were performed on the rotator. Membranes were washed with 5 ml TBS-T (TBS with 0.1% Tween), and incubated overnight at 4˚C with the same primary antibodies as used in the immunohistochemical (main text) and immunofluorescence staining (see above): A1AT rabbit polyclonal antibody and REG4 goat polyclonal antibody were diluted at 1:5000 and 1:1000 in 5 ml blocking buffer, respectively. Membranes were washed in TBS-T, incubated for 60 minutes with donkey anti-goat (for REG4) or goat anti-rabbit (Dako) (for A1AT), and washed again. To visualize the bands on the membrane, 500 µL of each the two solutions of Western Lightning Plus-ECL (PerkinElmer) was added on parafilm (Bemis, United States of America). The membrane was shortly dried on Whatman chromatography paper (GE Healthcare), placed on this parafilm and incubated for 5 minutes. The membrane was placed in a transparent plastic cover and ChemiDoc™ Touch Imaging System (Bio-Rad Laboratories) was used to image the bands on the membrane.
